# To detect myocardial ischemic zones on CMR in relation to degree of coronary artery stenosis and to correlate with major adverse cardiac events in 9 month follow up period

**DOI:** 10.1186/1532-429X-15-S1-E64

**Published:** 2013-01-30

**Authors:** Mahesha M Bannur, Amogh K Mallikrajunappa

**Affiliations:** 1 Radiology, Viikarm Hospital -Jeshta, Mysore, India

## Background

Perfusion-cardiac magnetic resonance (CMR) is generally accepted to assess myocardial ischemia non-invasively by assessing the ischemia noninvasively with moderate coronary artery stenosis and follow up of these patients will helps in prognostic estimation.

## Methods

Retrospective study of 68 patients with moderate coronary artery stenosis (50-70%) underwent stress and viability CMR in the period from January 2010 to December 2011. Patients are referred by cardiologists and physicians after coronary angiogram for further evaluation.

Cardiac MR done with state-of-the-art MR equipment Magnetom Avanto 1.5 T (Siemens) by using stress and viability sequences.

Follow up of these patients are done for major adverse cardiac events -MACE for 9 months.

## Results

Of 68 patients with CAD, 94 segments of moderate coronary artery stenosis are found. Stress CMR shows evidence of ischemia in 54 segments & infarcts in 4 patients.

Follow up these patients for major adverse cardiac events in 9 months , 18 patients had MI , 6 patients had unstable angina and 5 patients with LV dysfunction

## Conclusions

Stress CMR analyses patients with moderate coronary artery stenosis for ischemia and predicts the development of major adverse cardiac events

## Funding

No funding taken.

**Figure 1:**
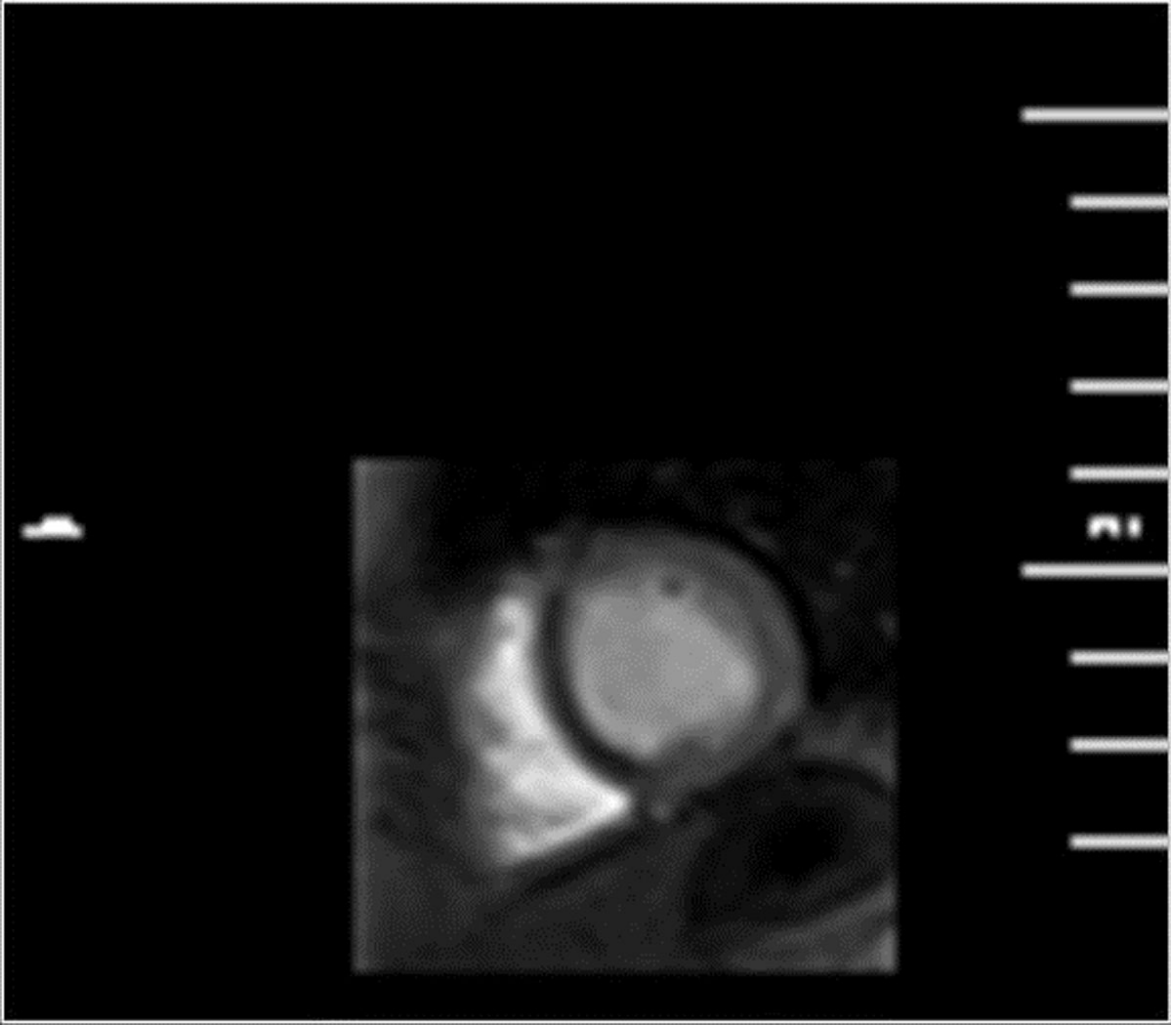
Adenosine stress MR - perfusion defect - LAD territory

**Figure 2:**
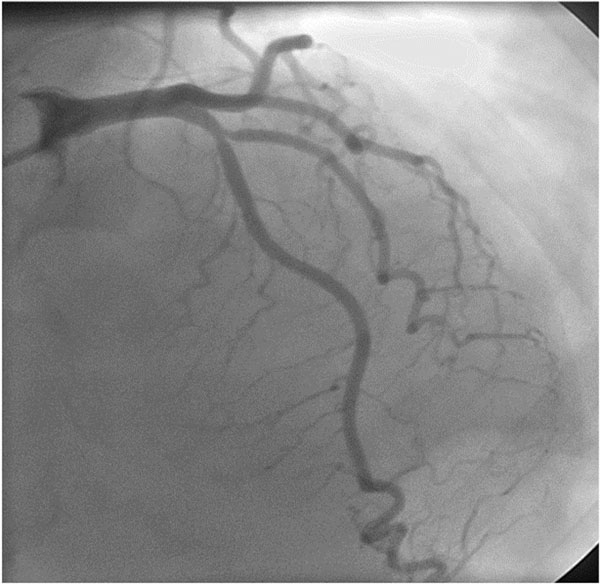
CAG - moderate LAD narrowing

